# Measurement-Based Quantum Correlations for Quantum Information Processing

**DOI:** 10.1038/s41598-020-59220-y

**Published:** 2020-02-12

**Authors:** Uman Khalid, Junaid ur Rehman, Hyundong Shin

**Affiliations:** Department of Electronic Engineering, Kyung Hee University, Yongin-si, 17104 Korea

**Keywords:** Quantum information, Quantum metrology

## Abstract

Measurement-based quantum correlations (MbQCs) depend on how strongly an observer perturbs the unobserved system. This distinctive property differentiates MbQCs from traditional quantum correlations such as entanglement and discord. We utilize MbQCs to elucidate quantum information processing capabilities in quantum computation and quantum state discrimination. We show that MbQCs exist more generally than entanglement and discord in optimal assisted quantum state discrimination and in a deterministic quantum computation with a single qubit. We also propose an MbQC-based dimension witness and analyze it in different noisy and noiseless scenarios.

## Introduction

Quantum resources are key ingredient to ensure quantum supremacy in quantum information processing tasks. The demistification of the role played by quantum correlations as a resource in quantum information processing is still under progress. To date, entanglement and discord type quantum correlations have been tested for its application in optimal assisted state discrimination, deterministic quantum computation and device-independent quantum information processing (DIQIP).

Entanglement and discord had been considered essential in quantum state discrimination and quantum computation tasks^1–5^. However, quantum dissonance was found to outcast entanglement and discord in terms of existence in optimal assisted quantum state discrimination method introduced by Roa, Retamal and Alid-Vaccarezza (RRA scheme) and deterministic quantum computation with a single qubit (DQC1) protocol^6–8^. This lead to further investigation of the key resources available for quantum information processing tasks. For example, super quantum discord exists more generally in RRA scheme than entanglement and discord^9^. Moreover, not all quantum correlations are resourceful in mixed-state computational circuits. For instance, nonclassical correlations induced by disturbance under measurement, i.e., measurement-induced disturbance (MID) are useful while the ones under unitary operations, i.e., locally noneffective unitary operation (LNU) provide no relative advantage since it vanishes asymptotically as system size grows^10^. This lead to a conjecture that there may exist quantum algorithms possessing exponential or polynomial advantage over classical counterparts without exploiting quantum entanglement as a resource^11,12^. Moreover, the accuracy achievable in DQC1 is related to the quantum coherence, which is the most basic ingredient of quantumness in a system^13^.

The dimensionality of a quantum system is also regarded as a resource in quantum information processing. Testing the Hilbert space dimension is relevant to security proofs of quantum key distribution (QKD) and realization of quantum random number generators (QRNG)^14,15^. The concept of dimension witness has been studied in the context of Bell inequalities, quantum random access codes, quantum observable, prepare-and-measure scenario, and in prepare-and-distribute scenario^16–20^. Single qubit probe indirect measurements also capacitate the exact dimension estimation of a quantum system provided all the entailed qubits are correlated for a preset interaction model^21^. Recently, the connection between dimension witness and quantum correlations in a high dimensional quantum system has been explored^22^. For example, ${\mathscr{P}}$-concurrence is both a dimension witness and an entanglement measure for high dimensional entangled systems but does not hold these properties for unentangled quantum states^23^. Similarly, super quantum discord arises from the statistics of weak measurement^24^. Weak measurement has significant applications in multi-observer dimension witnesses for DIQIP^25^. Furthermore, quantum witnesses also provide bound on the Hilbert-space dimension of an entangled quantum system^26^.

There exists measurement-based quantum correlations (MbQCs) that utilize strong perturbation effects induced by local von Neumann measurements. These quantum correlations are different from quantum entanglement, quantum discord and other known traditional quantum correlation measures^27^. Recently, MbQCs are discovered to be relatively more resourceful than quantum entanglement and super quantum discord in the field of mixed state quantum metrology^28^.

In this paper, we study the role played by MbQCs in RRA scheme, DQC1 protocol and dimension witness for DIQIP in an inspiration to contribute towards the resource theory of quantum information processing. Specifically, this work provides answers to: i) do MbQCs exist more generally in RRA scheme and DQC1 protocol, and ii) what is the necessary minimum dimension corresponding to the degree of MbQCs. In the following, we first introduce the MbQC measure for arbitrary dimensional bipartite mixed states. Then, we discuss the existence of MbQCs in an optimal state discrimination task and in quantum computation task. Finally, we introduce the MbQC-based dimension witness.

## Results

### Measurement-based quantum correlation

A quantum correlation measure for an arbitrary bipartite state ${\rho }^{AB}$ was defined in^27^ as

1 $${C}_{Q}({\rho }^{AB})=\mathop{{\rm{\sup }}}\limits_{\{{\Pi }_{y}^{A}\}}\sum _{y}{p}_{y}{\Vert {\rho }^{B}-{\rho }_{y}^{B}\Vert }_{2}^{2},$$

where $\{{\Pi }_{y}^{A}\}$ characterize local von Neumann measurement, ${\Vert T\Vert }_{2}={[{\rm{tr}}({T}^{\dagger }T)]}^{\mathrm{1/2}}$ is the Hilbert-Schmidt norm, and the normalizing factor ${p}_{y}={\rm{tr}}(({\Pi }_{y}^{A}\otimes {I}^{B}){\rho }^{AB}({\Pi }_{y}^{A}\otimes {I}^{B}))$. It quantifies the distance between the pre measurement reduced state ${\rho }^{B}={{\rm{tr}}}_{A}({\rho }^{AB})$ and the post measurement reduced state ${\rho }_{y}^{B}=\frac{1}{{p}_{y}}{{\rm{tr}}}_{A}(({\Pi }_{y}^{A}\otimes {I}^{B})\,{\rho }^{AB}({\Pi }_{y}^{A}\otimes {I}^{B}))\,$. ${\rho }_{y}^{B}$ can be regarded as Bob’s density matrix conditioned on the measurement result $y$ on Alice’s side. However, Hilbert-Schmidt distance is not a valid measure to quantify quantum correlations due to its nonmonotonicity under noisy maps^29,30^. Therefore, ${C}_{Q}({\rho }^{AB})$ was revised to introduce the measurement-based quantum correlation measure as^28^

2 $$Q({\rho }^{AB})=\mathop{{\rm{\sup }}}\limits_{\{{\Pi }_{y}^{A}\}}\sum _{y}{p}_{y}{\mathscr{D}}({\rho }^{B},{\rho }_{y}^{B}),$$

where ${\mathscr{D}}({\rho }^{B},{\rho }_{y}^{B})$ is the distance between Bob’s pre measurement state and post measurement conditional state. This distance metric can be any valid measure such as trace distance, Hellinger metric, and Bures metric^31^. Recall that for any two quantum states $\rho$ and $\sigma$, Hilbert-Schmidt distance, trace distance, Hellinger metric, and Bures metric are defined as $\sqrt{{\rm{tr}}{(\sqrt{\rho }-\sqrt{\sigma })}^{2}}$, $\frac{1}{2}\,{\rm{tr}}\sqrt{{(\rho -\sigma )}^{\dagger }(\rho -\sigma )}$, ${\rm{tr}}\,{(\sqrt{\rho }-\sqrt{\sigma })}^{2}$ and $\sqrt{2-2\,{\rm{tr}}\sqrt{\sqrt{\rho }\sigma \sqrt{\rho }}}$, respectively. $Q({\rho }^{AB})$ satisfies the requirements of a valid measure of quantum correlations^28,32^. That is, (i) $Q({\rho }^{AB})\ge 0$, with equality only for product states; (ii) $Q({\rho }^{AB})=Q(({U}^{A}\otimes {U}^{B}){\rho }^{AB}({U}^{A}\otimes {U}^{B}))$, i.e., invariant under local unitary operations; and (iii) $Q({\rho }^{AB})$ is monotonically nonincreasing under noisy maps.

### MbQCs in optimal assisted quantum state discrimination

In this section, we show that the MbQCs exist more generally than quantum discord and entanglement in optimal state discrimination tasks. To this end, we consider an example with regards to optimal state discrimination method of RRA scheme where such trend can be found for the case of minimum error probability^6,9^.

In the following, we first describe the RRA scheme. Consider two nonorthogonal states ${|{\psi }_{0}\rangle }^{B}$ and ${|{\psi }_{1}\rangle }^{B}$ with *a priori* probabilities ${p}_{0}$ and ${p}_{1}$, where the superscript $B$ denotes the system label. This principle qubit system is coupled with an auxiliary qubit system $A$ in the known initial state ${|{\mathscr{X}}\rangle }^{A}$ having orthonormal basis $\{{|0\rangle }^{A},{|1\rangle }^{A}\}$. This coupling action is described by a joint unitary transformation *U* as

$$\begin{array}{c}U{|{\psi }_{0}\rangle }^{B}{|{\mathscr{X}}\rangle }^{A}=\sqrt{1-|{\alpha }_{0}{|}^{2}}{|+\rangle }^{B}{|0\rangle }^{A}+{\alpha }_{0}{|0\rangle }^{B}{|1\rangle }^{A},\\ U{|{\psi }_{1}\rangle }^{B}{|{\mathscr{X}}\rangle }^{A}=\sqrt{1-|{\alpha }_{1}{|}^{2}}{|-\rangle }^{B}{|0\rangle }^{A}+{\alpha }_{1}{|0\rangle }^{B}{|1\rangle }^{A},\end{array}$$

where $|\pm \rangle =(|0\rangle \pm |1\rangle )/\sqrt{2}$ are the distinguishable orthonormal states of the system and the coefficients ${\alpha }_{0}$ and ${\alpha }_{1}$ are chosen such that $\langle {\psi }_{0}|{\psi }_{1}\rangle ={\alpha }_{0}^{\star }{\alpha }_{1}=\alpha$ is the fixed a priori overlap. This coupling leads the composite state to be

3 $${\rho }_{|{\alpha }_{0}|}^{BA}={p}_{1}U(|{\psi }_{1}\rangle \langle {\psi }_{1}|\otimes |{\mathscr{X}}\rangle \langle {\mathscr{X}}|){U}^{\dagger }+{p}_{0}U(|{\psi }_{0}\rangle \langle {\psi }_{0}|\otimes |{\mathscr{X}}\rangle \langle {\mathscr{X}}|){U}^{\dagger }\mathrm{}.$$

This expression indicates the presence of some quantum correlations between the principle system and auxiliary qubit depending on joint transformation. The initial states $|{\psi }_{0}\rangle$ or $|{\psi }_{1}\rangle$ can be discriminated unambiguously by performing von Neumann measurement in the basis $\{{|0\rangle }^{A},{|1\rangle }^{A}\}$ of the auxiliary system $A$. The successful detection can be achieved if auxiliary system is projected onto the state ${|0\rangle }^{A}$. RRA scheme can be termed as an assisted discrimination via local von Neumann measurement on auxiliary qubit A (right system) to distinguish two nonorthogonal states of principle qubit system B (left system). Therefore, any quantum correlation quantified via local measurement on principle system becomes an irrelevant resource in assisted state discrimination (optimal/non optimal)^7^. In the considered configuration of composite system, left and right discord are defined via measurement on principle and auxiliary system, respectively. Here, left discord becomes irrelevant and right discord becomes necessary. However, left discord becomes crucial and right discord is completely unnecessary upon exchange of the subsystems in the composite system. This leads to the proposition that quantum discord becomes an inconsistent resource in state discrimination method of RRA scheme, due to its observer dependency. On the other hand, MbQCs are defined as the distance between density matrices of the principle system, before and after performing local von Neumann measurement on auxiliary system. That is, MbQCs only depend on how strongly a measurement device acting on auxiliary system, can perturb the principle system. Hence, MbQC is the truly relevant resource that is consumed in such information processing task.

In the following we show that MbQCs are nonzero in the region where both entanglement and left quantum discord do not exist in the optimal case. Note that the left quantum discord as well as quantum entanglement in state (3) vanish simultaneously only for $\alpha \ge 0$; ${p}_{0}={p}_{1}=\mathrm{1/2}$; and $|{\alpha }_{0}|=|{\alpha }_{1}|=\sqrt{|\alpha |}=\sqrt{\alpha }$^7^. To this end, we use the parameterized form of the state (3) in terms of the only free variable, i.e., ${\alpha }_{0}=\zeta$, and show the trend of MbQC against this parameter^9^. The parameterized form of (3) with aforementioned constraints is

4 $${\rho }^{BA}=\frac{1-{\zeta }^{2}}{2}(I\otimes |0\rangle \langle 0|)+|0\rangle \langle 0|\otimes ({\zeta }^{2}|1\rangle \langle 1|+\frac{\zeta \sqrt{1-{\zeta }^{2}}}{\sqrt{2}}(|0\rangle \langle 1|+|1\rangle \langle 0|))\mathrm{}.$$

The optimal probability of successfully discriminating ${|{\psi }_{0}\rangle }^{B}$ from ${|{\psi }_{1}\rangle }^{B}$ simplifies to^6^

5 $${P}_{{\rm{success}}}=1-{\zeta }^{2}\mathrm{}.$$

**Figure 1:**
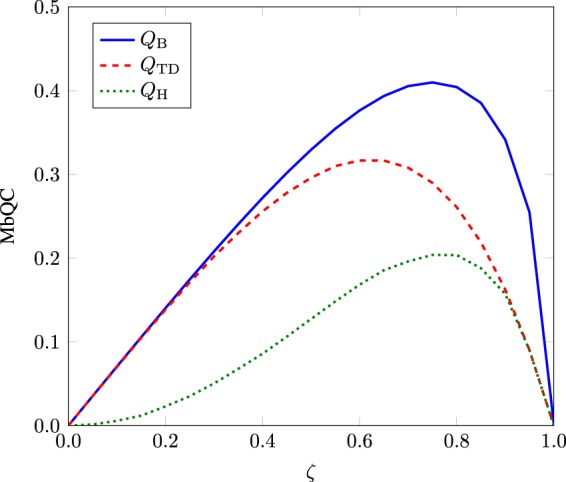
MbQCs in state (4) for Bures distance ${Q}_{{\rm{B}}}$, trace distance ${Q}_{{\rm{TD}}}$ and Hellinger distance ${Q}_{{\rm{H}}}$ as a function of $\zeta$.

For $\zeta =0$, the states are perfectly discriminated whereas the discrimination between two states becomes impossible for $\zeta =1$. For optimal measurement setting, the behavior of MbQCs for Bures distance ${Q}_{{\rm{B}}}$, trace distance ${Q}_{{\rm{TD}}}$ and Hellinger distance ${Q}_{{\rm{H}}}$ is shown in Fig. 1. MbQCs are nonvanishing for the specified range of $\zeta$. Note that, quantum entanglement and left quantum discord are zero for this particular region. The upper and lower bounds to MbQC can also be computed analytically in terms of fidelity^33^. Hence, MbQCs exist more universally in optimal assisted quantum state discrimination tasks than quantum entanglement and left quantum discord. However, right quantum discord is present in this scheme and so is super quantum discord^9^.

### MbQCs in deterministic quantum computation

**Figure 2:**
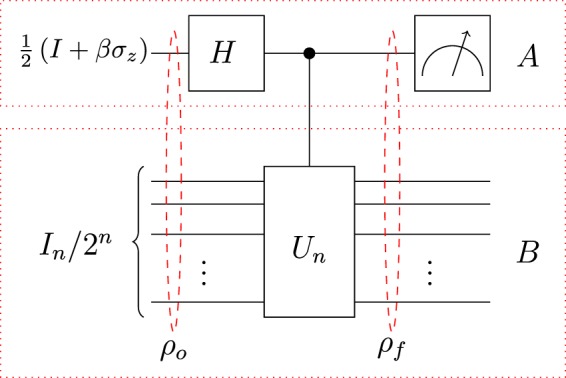
DQC1 Protocol: The model consists of one qubit with parameter *β*, accompanied by a register of *n* maximally mixed qubits. For computation, Hadamard operator acts on top qubit whose output state controls *n* qubit unitary ${U}_{n}$. Expectation value measurements on single qubit *A* provide estimates for normalized trace of unitary.

In this section, we characterize the role of MbQCs in a quantum information processor model namely, deterministic quantum computation with a single qubit (DQC1)^34^. The protocol efficiently estimates the normalized trace of unitary matrix ${U}_{n}$ given by $\tau ={\rm{tr}}({U}_{n}){\mathrm{/2}}^{n}$ by performing measurements on single qubit system $A$ as shown in Fig. 2. The averages of the obtained binary values from measurements ${\sigma }_{x}$ and ${\sigma }_{y}$ on $A$ provide estimates for the real and imaginary parts given as ${\tau }_{R}={\rm{Re}}(\tau )$ and ${\tau }_{I}={\rm{Im}}(\tau )$, respectively. The circuit of Fig. 2 transforms an *n* + 1-qubit highly mixed input state

6 $${\rho }_{o}=\frac{1}{2}(I+\beta {\sigma }_{z})\otimes \frac{{I}_{n}}{{2}^{n}},$$

to the final state

7 $${\rho }_{f}=\frac{1}{{2}^{n+1}}(|0\rangle \langle 0|\otimes {I}_{n}+|1\rangle \langle 1|\otimes {I}_{n}+\beta |1\rangle \langle 0|\otimes {U}_{n}+\beta |0\rangle \langle 1|\otimes {U}_{n}^{\dagger }),$$

where $\beta$ determines the purity of the single qubit system $A$ in ${\rho }_{o}$ as $(1+{\beta }^{2})\mathrm{/2}$.

This final state exhibits small entanglement content across any bipartite split that vanishes for $\beta \le \mathrm{1/2}$^4^. Additionally, this state always remains separable across the split $A|B$ shown in Fig. 2. The amount of nonclassical correlations in this configuration depends on the controlled unitary represented as $|1\rangle \langle 1|\otimes {U}_{n}+|0\rangle \langle 0|\otimes {I}_{n}$. If the corresponding unitary has product eigenbasis, then it produces a trivial map, which enables efficient classical simulation of such quantum circuits (e.g. Clifford group evolution)^35,36^. However, there exist unitaries ushering a nontrivial map that generates nonclassical correlations, as quantified by their discording power^37^. Efficient classical simulation of such maps is considered to be impossible^38,39^. Observance of nonclassical correlations in DQC1 circuit under such unitaries induced the intuition that all those DQC1 circuits resulting in the generation of nonclassical correlations are not classically simulable^40^. Hence, the presence of nonclassical correlations in DQC1 appeared as a signature of quantum speed-up^41^.

**Figure 3:**
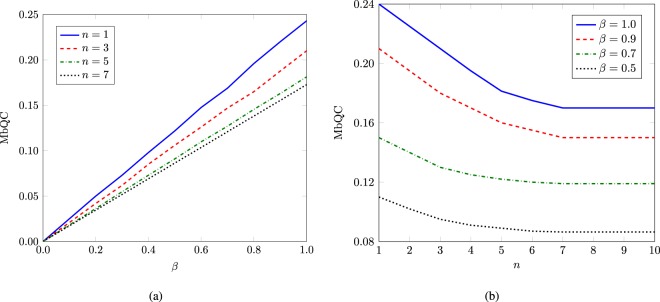
MbQCs for trace distance ${Q}_{{\rm{TD}}}$ as a function of (**a**) *β*, and (**b**) *n*. Entanglement does not exist for this particular case.

**Figure 4:**
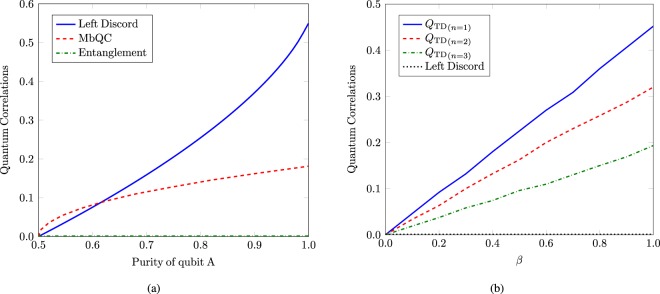
MbQCs for trace distance ${Q}_{{\rm{TD}}}$ and left quantum discord as a function of (**a**) purity of qubit A, given by ${\rm{tr}}({\rho }_{A}^{2})=(1+{\beta }^{2})\mathrm{/2}$, for Haar random unitary having ${\rm{tr}}({U}_{n}^{2}{\mathrm{)/2}}^{n}\approx 0$, and (**b**) $\beta$ for random Hermitian unitary having ${\rm{tr}}({U}_{n}^{2}{\mathrm{)/2}}^{n}\approx 1$.

Now, we numerically evaluate the average values of MbQCs for this configuration with over 1000 realizations of random unitaries generated uniformly according to the Haar measure^42^. By increasing such realizations, average values of MbQCs nicely converge to a constant value after atleast 1000 realizations. These random unitaries benchmark the precedence of quantum computation over classical computation^43^. For optimal measurements, MbQCs are plotted as a function of *β* as well as for *n*. MbQCs increase linearly with *β* for a fixed number of qubits in the maximally mixed state of system *B*. MbQCs decrease with increasing the size of system *B* but do not vanish completely in the asymptotic limit. Hence, MbQCs become independent of the size of system *B* for larger values of *n* as shown in Fig. 3. The presence of MbQCs in the DQC1 protocol outcast quantum entanglement and right quantum discord as a figure of merit of nonclassical correlations in such a quantum information processor. However, left quantum discord is present in the considered bipartite split in DQC1 circuit^5^. The comparative behavior exhibited by left quantum discord and MbQC is depicted in Fig. 4(a). Left quantum discord increases rapidly whereas MbQCs show relatively slow increase, as a function of purity of qubit A. MbQCs are larger than left discord for $\beta \le \mathrm{1/2}$, and capture actual nonclassical correlations in the absence of entanglement for $\beta \le \mathrm{1/2}$. Left discord never vanishes for the case of Haar random unitaries. But there exists special random Hermitian unitaries that lead to vanishing left quantum discord^44^. Such unitaries are represented in the form ${U}_{n}={e}^{\iota \theta }H$, where $\theta \in [\mathrm{0,}\pi ]$ and ${H}^{2}=I$ is a binary observable^45^. One candidate of such involutory operators is a Householder matrix $H=I-2P$, where ${P}^{2}=P$ is an orthogonal projector. MbQCs do not vanish for such Hermitian unitaries as shown in Fig. 4(b). Hence, MbQCs outcast discord in terms of existence in DQC1.

### Quantum dimension witness

**Figure 5:**
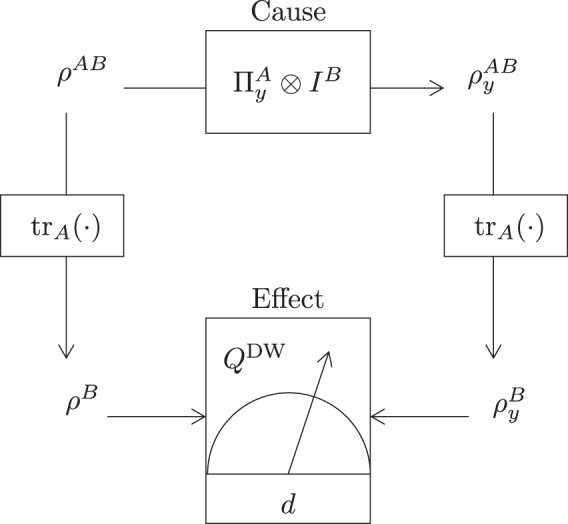
A cause-and-effect scenario where the measurement is performed by a third independent party on Alice’s part. After receiving the measurement results, Bob can locally quantify the perturbation caused by this measurement as a difference of density operators with known and unknown measurement results.

In this section, we introduce an MbQC-based quantum dimension witness for DIQIP. For this, the local measurement setting is extended to usher in a cause-and-effect scenario that yields the maximally perturbed post measurement Bob’s conditional state ${\rho }_{y}^{B}$ as shown in Fig. 5. This quantum effect can be described as the change in the local state of Bob as a result of conditioning on the measurement result of Alice’s part. This effect enables the certification of the dimensions of a system merely by sensing the change in Bob’s state. Thus, the perturbation effect is utilized to define an MbQC witness as

8 $${Q}^{{\rm{DW}}}=\mathop{{\rm{\max }}}\limits_{\{{\Pi }^{B}\},y}|{\rm{tr}}\{{\Pi }^{B}({\rho }^{B}-{\rho }_{y}^{B})\}|,$$

where the maximization is performed with respect to all projectors $\{{\Pi }^{B}\}$, and measurement outcomes $y$. Clearly, MbQC witness is zero for product (uncorrelated) states which are immune to perturbation. However, MbQC certification is possible for any nonproduct (correlated) state. The witness follows the following chain of inequalities

9 $${Q}^{{\rm{DW}}}\le {{\mathscr{D}}}_{{\rm{TD}}}({\rho }^{B},{\rho }_{{y}_{{\rm{\max }}}}^{B})=\frac{1}{2}{\Vert {\rho }^{B}-{\rho }_{{y}_{{\rm{\max }}}}^{B}\Vert }_{1}\le 1-\frac{1}{d},$$

where ${\Vert T\Vert }_{1}={\rm{tr}}{({T}^{\dagger }T)}^{\mathrm{1/2}}$ is the trace norm. The first inequality follows from the fact that trace distance between pre measurement reduced state and post measurement reduced state is equal to the probability difference exhibited by them under same measurement [^46^, Lemma 9.1.1]. First equality arises from the definition of trace distance while the second inequality arises from the convexity of trace distance which saturates for bipartite qudits in which Bob’s pre measurement state is maximally mixed, i.e., ${\rho }^{B}={I}_{d}/d$ and post measurement conditional state is pure^47^. MbQC witness behaves as a dimension witness since ${Q}^{{\rm{DW}}}$ corresponds to the minimum dimension required to exhibit ${Q}^{{\rm{DW}}}$ for a given quantum system. In other words, the maximization of MbQCs enables the witnessing of quantum dimension. Therefore, (9) allows MbQC witness to operate as a dimension witness as

10 $$d\ge \frac{1}{1-{Q}^{{\rm{DW}}}}\mathrm{}.$$

Now, we compare the proposed dimension witness with other dimension witnessing strategies that also stem from the relation between quantum state discrimination and dimension witness, namely prepare-and-measure scenario and prepare-and-distribute scenario. For the dimension witness based on prepare-and-measure scenario to be meaningful, the number of preparations of the test system must be higher than its dimension^19^. Contrary to this, ${Q}^{{\rm{DW}}}$ acts as a simpler and resource efficient dimension witness in the sense that the two reduced density matrices are related to each other via local measurement in a cause-and-effect configuration. The performance of dimension witness based on prepare-and-distribute scenario deteriorates with decreasing entanglement^20^. However, ${Q}^{{\rm{DW}}}$ is immune to such anomaly. For instance, the quantum states saturating the relation (10) need not to be either entangled or pure, since both maximally correlated unentangled state ${\sum }_{i=0}^{d-1}\mathrm{1/}d(|i\rangle {\langle i|}^{A}\otimes |i\rangle {\langle i|}^{B}),$ and the maximally entangled state ${\sum }_{i\mathrm{=0}}^{d-1}\mathrm{1/}\sqrt{d}\,{|i\rangle }^{A}{|i\rangle }^{B}$ maximize ${Q}^{{\rm{DW}}}$ for given dimension and possess maximally mixed marginals. This benchmarks the proposed dimension witness from existing entanglement based dimension witness in terms of the scope and dimension witnessing capabilities. Now, we illustrate the robustness of ${Q}^{{\rm{DW}}}$ in noise by exemplifying the application of amplitude damping and depolarizing channel on maximally entangled initial states.

#### Robustness under noise

To demonstrate the robustness of dimension witness under noise, consider a bipartite qudit GHZ state as

11 $${|\Psi \rangle }^{AB}=\mathop{\sum }\limits_{k\mathrm{=1}}^{d}\frac{1}{\sqrt{d}}{|k\rangle }^{A}{|k\rangle }^{B}\mathrm{}.$$

The quantum state undergoes decoherence when interacting with the environment. The operator-sum representation of such noisy maps is

12 $${|\Psi \rangle }^{AB}\to {\rho }^{AB}=\sum _{k}{K}_{k}|\Psi \rangle {\langle \Psi |}^{AB}{K}_{k}^{\dagger },$$

where ${K}_{k}$ are the Kraus operators satisfying ${\sum }_{k}{K}_{k}^{\dagger }{K}_{k}=I$. The Kraus operators for the amplitude damping channel for $d=2$ are given by

13 $${K}_{0}=|0\rangle \langle 0|+\sqrt{1-p}|1\rangle \langle 1|,\,{K}_{1}=\sqrt{p}|0\rangle \langle 1|,$$

where *p* is the strength of amplitude damping noise. For $d=3$, the Kraus operators are given by

14 $$\begin{array}{c}{K}_{0}=|0\rangle \langle 0|+\sqrt{1-p}|1\rangle \langle 1|+(1-p)|2\rangle \langle 2|,\\ {K}_{1}=\sqrt{p}|0\rangle \langle 1|+\sqrt{2p(1-p)}|1\rangle \langle 2|,\\ {K}_{2}=p|0\rangle \langle 2|\mathrm{}.\end{array}$$

**Figure 6:**
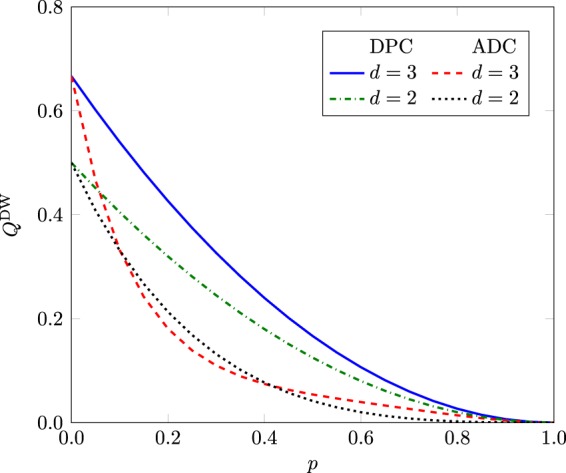
${Q}^{{\rm{DW}}}$ as a function of parameter $p$ for GHZ state.

The Kraus operators for the depolarizing channe l for $d=2$ are

15 $${K}_{0}=\sqrt{1-\frac{3p}{4}}{I}_{2},\,{K}_{1}=\sqrt{\frac{p}{4}}{\sigma }_{x},\,{K}_{2}=\sqrt{\frac{p}{4}}{\sigma }_{y},\,{K}_{3}=\sqrt{\frac{p}{4}}{\sigma }_{z},$$

where ${I}_{2}$ is the 2 × 2 identity matrix, $p$ is the strength of depolarization noise, and ${\sigma }_{x},{\sigma }_{y},{\sigma }_{z}$ are Pauli spin operators. For $d=3$, Kraus operators are given as^48^

16 $$\begin{array}{c}{K}_{0}=\sqrt{1-p}{I}_{3},\,{K}_{1}=\sqrt{\frac{p}{8}}Y,\,{K}_{2}=\sqrt{\frac{p}{8}}Z,\\ {K}_{3}=\sqrt{\frac{p}{8}}{Y}^{2},\,{K}_{4}=\sqrt{\frac{p}{8}}YZ,\,{K}_{5}=\sqrt{\frac{p}{8}}{Y}^{2}Z,\\ {K}_{6}=\sqrt{\frac{p}{8}}Y{Z}^{2},\,{K}_{7}=\sqrt{\frac{p}{8}}{Y}^{2}{Z}^{2},\,{K}_{8}=\sqrt{\frac{p}{8}}{Z}^{2},\end{array}$$

where *Y* and *Z* are respectively given by

$$Y=[\begin{array}{ccc}0 & 1 & 0\\ 0 & 0 & 1\\ 1 & 0 & 0\end{array}],\,Z=[\begin{array}{ccc}1 & 0 & 0\\ 0 & \omega  & 0\\ 0 & 0 & {\omega }^{2}\end{array}],$$

with $\omega ={e}^{2\pi \iota \mathrm{/3}}$. The behavior of ${Q}^{{\rm{DW}}}$ under depolarizing noise and amplitude damping noise is shown in Fig. 6. It has been shown that dimension witness serves well for the depolarizing noise but in case of the amplitude damping noise, energy relaxations obscures testing of the dimensions of a decohered GHZ state.

## Conclusion

We found that the resourceful quantum correlations for quantum information processing tasks depend not only on a specific observer, e.g., in left or right quantum discord and a weak observer such as super quantum discord, but also on the fact that how strongly an observer can disturb the unobserved system such as in MbQCs. We showed that in RRA scheme, MbQCs outcast entanglement and discord in terms of existence. Similarly, MbQCs can be regarded as a resource in applications of DQC1 circuit, which employs MbQCs. Furthermore, we proposed an MbQC-based dimension witness, which tests and certifies the dimension of a quantum system. The proposed dimension witness outperforms the entanglement-based dimension witnesses due to its reliance not on entanglement but on MbQCs, which encompass entanglement. However, such witnesses cannot discriminate between quantum and classical systems. These results are useful in the fields of quantum computation, quantum information processing and quantum detection and estimation tasks.
